# Acquired Factor V Inhibitor After Antibiotic Therapy: A Clinical Case Report and Review of the Literature

**DOI:** 10.7759/cureus.9481

**Published:** 2020-07-30

**Authors:** Alexander R Chartier, Conor J Hillert, Harpreet Gill, Pinky Jha

**Affiliations:** 1 Internal Medicine, Medical College of Wisconsin, Wauwatosa, USA; 2 Internal Medicine, Medical College of Wisconsin, Brookfield, USA

**Keywords:** acquired factor v inhibitor, antibiotics, cephalosporins, warfarin, coagulopathy

## Abstract

Acquired factor V inhibitor (aFVi) is an exceptionally rare hematologic condition that can range from incidental laboratory abnormalities to life-threatening hemorrhage. Bovine thrombin was formerly the most common cause of this condition; however, the decreased use of bovine thrombin in surgical procedures has led to a shift in the cause of aFVi toward antibiotic use and malignancies. Here we present a case of an 80-year-old Caucasian female on long-term warfarin therapy who presented with epistaxis and an elevated international normalized ratio, and a history of cephalosporin antibiotic use. We review the published literature beginning in 2016 to identify the evolving causes of aFVi. Additionally, we propose that stress-mediated immune regulation may contribute to antibody formation, preventing the interaction between factor V and the damaged phospholipid membranes. This case highlights the evolving causes of aFVi and should prompt physicians to consider this diagnosis in coagulopathies that do not correct with traditional therapies.

## Introduction

Acquired hemophilia is a rare and clinically challenging scenario as patient presentation can range from clinically asymptomatic laboratory abnormalities to life-threatening hemorrhage. Acquired factor V inhibitors (aFVis) were first identified by Hӧrder in 1955 and twice more by Ferguson et al. in 1957 and 1958 [1]. Since the initial reports, physicians have become aware of the associations between surgical procedures, antibiotics, immunosuppressants, and bovine serum proteins, and this rare condition. In their review of 105 previous cases in 1998, Knobl and Lechner found that from the identified factors, the majority of cases were secondary to the use of bovine thrombin as a hemostatic agent [1]. Bovine thrombin has largely been replaced since the FDA approval of recombinant human thrombins in both 2007 and 2008 [2]. The use of a less immunogenic thrombin has removed the largest cause of aFVi, further decreasing the incidence of this already exceedingly rare disease. Recently, there has been a shift in the associated causes of aFVi toward antibiotics, malignancies, and autoimmune disorders [3]. There is not an accepted mechanism as to how antibodies are formed against factor V (FV) in these situations. Two retrospective studies found that aFVi had an incidence of between 0.06 and 0.09 cases per one million people per year [4]. The true incidence of aFVi is likely higher due to the presence of asymptomatic carriers, which were found to represent between 19% and 31% of cases in literature reviews [3-5]. Here we present a case of a patient who was prescribed cephalosporin antibiotics after a podiatric procedure and subsequently presented with increased international normalized ratio (INR) and epistaxis and was found to have developed aFVi.

## Case presentation

An 80-year-old obese Caucasian female with a relevant past medical history significant for deep vein thromboses in the 1970s being managed with warfarin presented to the emergency department with epistaxis for 24 hours and INR > 10 on her home monitor. Prior to this, the patient denied any recent dose change to her warfarin or taking any additional doses. The patient denied any changes to diet or activity. The patient was seen by a podiatrist three weeks prior for a left hallux total nail removal and was prescribed a course of cephalexin 500 mg two times daily for seven days, which she completed.

The patient was well appearing on presentation in the emergency department (ED); she denied hematemesis, melena, or hematuria. The patient denied any changes to medications except for the recent cephalexin use. The patient denied any known family bleeding disorders. Physical exam was significant for dried blood in the nares, and blood was seen in the posterior oropharynx. Additionally, it was noted she had a large ecchymosis on her right breast. Vital signs were unremarkable in the ED. Lab results were significant for hemoglobin level of 14.1 g/dL (reference range: 11.3-15.1 g/dL), red blood cell count of 5.0 x 10^6^/μL (reference range: 3.7-5.2 x 10^6^/μL), white blood cell count of 8.2 x 10^3^/μL (reference range: 3.9-11.2 x 10^3^/μL), and platelet count 218 x 10^3^/μL (reference range: 165-366 x 10^3^/μL). A coagulation profile revealed INR, prothrombin time (PT), and activated partial thromboplastin time (APTT) above detectable limits (Table 1). In the ED, she was given 2.5 mg oral vitamin K. The patient was then admitted to the general medicine service for further evaluation and management.

**Table 1:** Selected Labs and Treatment of First Admission PO, oral administration; SQ, subcutaneous; PCC, prothrombin complex concentrate; PT, prothrombin time; INR, international normalized ratio; APTT, activated partial thromboplastin time; PT mix, prothrombin time mixing study; APTT mix, activated partial thromboplastin mixing time

Day of admission	0	1	2	3	4	5	6	7
Treatment	2.5 mg PO vitamin K	5 mg PO vitamin K	10 mg PO vitamin K, 10 mg SQ vitamin K, 10 mg IV vitamin K	PCC	100 mg PO prednisone	Rituximab 375 mg/m^2^ x 2.26 m^2^ (did not finish), 100 mg PO prednisone	Rituximab (finished), 100 mg PO prednisone	100 mg PO prednisone
PT (9.5-11.2 seconds)	>90	>90	>90	>90	>90	>90	>90	>90
INR	>10	>10	>10	>10	>10	>10	>10	>10
APTT (22-29 seconds)	>139				>139		>139	>139
PT mix (9.5-11.2 seconds)				44.7				
APTT mix (22.0-29.0 seconds)				84				
Thrombin time (0.0-23.9 seconds)					19.1			
Factor V inhibitor					30 inhibitor units			

On day 1 of hospital admission, the ecchymosis on the patient’s right breast was noted to be increasing in size from hospital day 0. On days 1 and 2 of admission, the patient was treated with multiple doses of oral and intravenous Vitamin K with no significant change in laboratory studies (Table 1). The patient continued to be hemodynamically stable. During this time, multiple ecchymoses on her right breast appeared, and the initial ecchymosis continued to increase in size. The hematology service was then consulted for further management.

On day 3 of hospitalization, mixing studies were ordered, which showed partial correction for both PT (corrected to 44.7 seconds [9.5-11.2 seconds]) and APTT (corrected to 84 seconds [22.0-29.0 seconds]). Factor VIII was 270 (64-189 %ACT). Factors II, VII, IX, and X were within normal limits. Disseminated intravascular coagulation (DIC) panel was negative, and thrombin time was within normal limits. FV activity was <5% (62-140%), and a standard Bethesda assay confirmed aFVi by the presence of an FV inhibitor with 30 inhibitor units (Table 1). The patient continued to be stable hemodynamically and had unremarkable vital signs. There were increased ecchymoses bilaterally on her upper and lower extremities and at the sites of IV access. Treatment was begun with prednisone 100 mg PO (oral) daily on day 4 of hospitalization and rituximab 375 mg infusion was begun on day 5, with the plan for weekly infusions for a total of four weeks. The first infusion was begun inpatient, and the patient began to show symptoms of an infusion reaction; therefore, the infusion was stopped and finished the next day. The patient was discharged from the hospital hemodynamically stable with INR > 10, PT > 90 seconds (9.5-11.2 seconds), and APTT > 139 seconds (22-20 seconds) with plans to see hematology outpatient and continue with rituximab infusions weekly on an outpatient basis.

Since the first admission, the patient has been re-admitted twice, once for melenic stools and a second time for pulmonary embolism. The first readmission was four days following the patient’s initial discharge. Her labs on admission were significant for a hemoglobin level of 6.3 g/dL, red blood cell count of 2.2 x 10^6^/μL, white blood cell count of 18.4 x 10^3^/μL, and platelet count of 378 x 10^3^/μL, and coagulation labs also were collected (Table 2). On day 1 of admission, the patient underwent an upper endoscopy, which showed no active source of bleeding. On day 4, FV activity was measured as 13% (62-140%). Labs at the time of discharge were obtained (Table 2). The patient was readmitted a second time for shortness of breath, which upon investigation was found to be a pulmonary embolism. On day 3, FV activity was at 106% (62-140%). She was discharged in stable condition on day 12 of admission after being treated symptomatically. The patient’s coagulation labs on admission and discharge were drawn (Table 3). The patient continues to see the hematology service in the outpatient clinic.

**Table 2:** Selected Coagulopathy Labs of Second Admission PT, prothrombin time; INR, international normalized ratio; APTT, activated partial thromboplastin time

Day of admission	0	7
PT (9.5-11.2 seconds)	62	12.7
INR	6.8	1.2
APTT (22-29 seconds)	77.7	-

**Table 3:** Selected Coagulopathy Labs of Third Admission PT, prothrombin time; INR, international normalized ratio; APTT, activated partial thromboplastin time

Day of admission	0	12
PT (9.5-11.2 seconds)	11.3	14.7
INR	1.1	1.4
APTT (22-29 seconds)	25.4	-

## Discussion

aFVi is a rare hematologic disorder with widely variable presenting symptoms. Commonly associated conditions are autoimmune disorders (13%), cancer (22%), and the use of antibiotics (42%) including beta-lactams, aminoglycosides (especially streptomycin), tetracyclines, and fluoroquinolones (especially ciprofloxacin) [3]. As patients can range from clinically asymptomatic to experiencing serious life-threatening bleeding diathesis, laboratory tests are critical to making the diagnosis of aFVi, and the interpretation of the tests can be difficult. The most common lab identification of aFVi is with grossly prolonged PT and APTT, which are only partially corrected with mixing studies. The inhibitor can then be confirmed through further laboratory testing. A number of treatments have been reported to reverse coagulopathy in case reports. Corticosteroids are typically used with success, and, in recent years, immunomodulating medications such as rituximab have also been utilized [3].

A literature search of previous reviews was conducted. In their 2010 systematic review, Franchini and Lippi reviewed cases of aFVi not associated with bovine protein. Of the 154 total cases identified beginning from 1955, the authors found 78 cases not associated with bovine thrombin [3]. Of these cases 42% (n = 33) were associated with antibiotics. Among antibiotics, the most represented were beta-lactams and aminoglycosides [3].

In a subsequent review, Boland and Shreenivas used a similar approach to examine cases from 2010 to 2016. Again, cases involving bovine thrombin were excluded to reflect the switch to human recombinant thrombin. Boland and Shreenivas found an additional 47 cases [5]. Infections were the most commonly implicated cause (n = 16), whereas antibiotics were the second most associated cause of aFVi (n = 15). The most common antibiotics implicated were beta-lactams. Combined with the previous study by Franchini and Lippi, the total number of cases up to 2016 not involving bovine thrombin is 125; and the most commonly reported association is antibiotic use, which is present in 38% of all cases [5].

A review of case reports in the previous five years was conducted. To provide continuity with both of the previous systematic reviews, cases involving bovine serum were excluded. Additionally, any cases included in the previous 125 reports were excluded. Cases were found by using the search terms “acquired factor v inhibitors” and “acquired coagulation inhibitors” on PubMed. Initially, 28 new cases were found from 26 published case reports. Of these, nine were included in the Boland and Shreenivas’s review and excluded from the table. Overall, 12 new cases were found on PubMed from 2016 to 2020 after removing congenital causes of FV deficiency and articles without full text availability (Table 4). The most common association was with antibiotics (5 out of 12), with beta-lactams being implicated in all antibiotic-related cases (Table 4).

**Table 4:** Compiled Literature of Acquired Factor V Inhibitors from 2016 to 2020 rFVIIa, recombinant factor VIIa; FPP, fresh frozen plasma

Study	Age (years)/gender	Hemorrhagic symptoms	Reported association	Treatment	Outcome
Andreadis et al. [6]	78/M	Ecchymosis of the left hemithorax	β-lactam, surgery	Steroids, rituximab, cyclophosphamide, rFVIIa	Remission (did not recheck inhibitor levels)
Nakata et al. [7]	72/F	None	unknown	Steroids, cyclophosphamide, plasma exchange	Remission
Quek et al. [8]	57/F	Knee hemarthrosis	Light chain myeloma	Bortezomib, cyclophosphamide, steroids	Remission
Fujita t al. [9]	82/M	Gum bleeding	Myelodysplastic syndrome	Steroids, platelet transfusions	Remission
Ogawa et al. [10]	87/M	Recurrent bleeding from hemodialysis sites	β-lactam, fluoroquinolone	Steroids, plasma exchange	Death due to aspiration pneumonia and general debility four months after second hospitalization
Taniwaki et al. [11]	78/M	None	β-lactam, fluoroquinolone, meropenem, micafungin, cefcapene pivoxil	Discontinued antibiotics	Remission
Li et al. [12]	1) 51/M 2) 78/M	1) Oral mucosal bleeding 2) Intracranial hemorrhage	1) β-lactam 2) Idiopathic	1) Steroids 2) FPP	1) Remission 2) Remission
Meidert et al. [13]	82/F	None	Idiopathic	FFP	No remission of inhibitor, but had no symptoms
Sakatoku et al. [14]	80/M	Nasal hemorrhage, hematuria	Prasugrel hydrochloride	Discontinued prasugrel, rFVIIa, steroids	Remission
Mihara et al. [15]	74/M	Subcutaneous hemorrhage on the left upper chest	Idiopathic	Steroids, cyclophosphamide	Remission
Yamanishi et al. [16]	84/M	Subcutaneous hemorrhage	β-lactam	Steroids	Died of respiratory/cardiac failure unrelated to coagulopathy

aFVi treatment involves two steps: first is to control symptomatic bleeding and second is to remove the inhibitor. Fresh frozen plasma (FFP), prothrombin complex concentrates (PCC), and platelet transfusions have been used to varying degrees of success [4]. In our case, we used vitamin K and PCC to control bleeding, which did not improve the patient's worsening ecchymoses. The use of PCC and FFP generally are thought to have low utility in treatment because of their low levels of FV. A trend in recent literature is the use of platelet transfusions as first-line treatment to control acute bleeding. The rationale behind this is that roughly 20% of a human’s FV is stored in the platelets and platelets have shown to be relatively unaffected by FV inhibitors, possibly by the distinct features of plasma and platelet FV [17]. Ang et al. showed the ability of platelet concentrates to normalize FV in 11 out of 16 cases, whereas Boland and Shreenivas showed 7 out of 12 had normalization of FV with platelet concentrates [4]. We found in our literature review that 1 out of 12 cases utilized platelet transfusions in their treatment plan, which resulted in remission in the single case (Table 4).

The second step in treatment is to remove the inhibitor, which can be done by a variety of methods. High-dose intravenous immunoglobulin (IVIG) has been shown to decrease anti-FV inhibitor titers [18]. Boland and Shreenivas showed that 88% of patients who received immunosuppression with corticosteroids alone achieved remission [5]. Rituximab has also been utilized in addition to corticosteroids, and there have been positive results, including our presented case. However, it is hard to ascertain if these results would have been equally obtained with steroids alone [5]. We found in our literature review that steroids were utilized in 9 out of 11 cases in which medication was given as part of treatment, whereas only one of the cases utilized rituximab (Table 4).

Theorized pathophysiology

A number of theories have been suggested for antibiotic-related aFVi. These include stress-based dysregulation of the immune system prior to antibiotic administration and simple homology between antibiotics and FV.

Prior research has indicated that the characteristics of the FVi (antibody) have been shown to best correlate with clinical bleeding risk rather than titers of the inhibitors. These characteristics include the inhibitor’s capacity to access FV stored in the alpha granules of platelets as well as to recognize the epitope in the C2 domain of the FV light chain [3]. Normally, FV acts by binding exposed procoagulant phospholipid-phosphatidylserine on activated platelets or endothelial cells through this C2 domain of the light chain [3]. This complex then binds to von Willebrand factor and enhances the coagulation process by activating factor X, which cleaves prothrombin to thrombin. Similarly, there is evidence of some acquired factor VIII inhibitors having inhibitors against the C2 domain of the light chain (as well as the A2 and A3 domains of the heavy chain), which suggests that there may be a similar mechanism causing both FVIII and FV inhibitor formation [19]. Interestingly, there is a strong body of evidence linking antibiotic classes (beta-lactams and aminoglycosides) strongly associated with acquired FV inhibitors to interactions with similar phospholipid residues [20]. In particular, many of the associated antibiotics are known to interact with phospholipids in the membranes of the bacterial cells they target. We suggest that in a heightened immunologic state of cancer, autoimmunity, or infection, the immune system may aberrantly produce antibodies against these antibiotics, which may themselves have some homology to the C2 region of the clotting factors’ light chains. However, further research into the inciting molecular and cyto-biologic mechanisms of aFVi formation is needed in light of many complex clinical presentations, a multitude of different associations, and a lack of in-depth antibody sequence analyses.

## Conclusions

aFVi is a rare and potentially fatal coagulopathy that is thought to be underreported in the literature. There are a limited number of cases reported on this condition, with variable causes. We report this case to increase awareness among practicing clinicians of this condition. Furthermore, we compiled a summary of aFVi cases published in the past five years to better characterize the common causes of this coagulopathy. Finally, we hypothesize that stress-mediated immune dysregulation may contribute to antibody formation blocking interactions between the C2 epitope of FV and the damaged phospholipid membrane residues. Further sequencing analysis of the antibodies and homology analyses must be performed to confirm or refute our hypothesized mechanism.
